# Label-Free Quantification of Bilirubin Using a Refractive Index-Insensitive Nanolaminate SERS Substrate

**DOI:** 10.3390/bios16050282

**Published:** 2026-05-14

**Authors:** Jiwon Yun, Inyoung Kim, Wonil Nam

**Affiliations:** 1 Department of Intelligent Robotics Engineering, Pukyong National University, Busan 48513, Republic of Korea; 202112150@pukyong.ac.kr; 2 Department of Statistics, Virginia Polytechnic Institute and State University, Blacksburg, VA 24061, USA; inyoungk@vt.edu; 3 Department of Electronic Engineering, Pukyong National University, Busan 48513, Republic of Korea

**Keywords:** surface-enhanced Raman spectroscopy, refractive index-insensitive, label-free, bilirubin quantification

## Abstract

Bilirubin is an important biomarker, where a small unbound fraction dissociated from albumin can cross the blood–brain barrier and induce neurotoxicity, such as kernicterus, at low nanomolar levels. Accurate detection of this low-level fraction remains challenging. Surface-enhanced Raman spectroscopy (SERS) enables label-free molecular detection; however, variations in the local refractive index (RI) at plasmonic hotspots can detune the resonance from the excitation wavelength, leading to signal fluctuations and limited quantitative reliability. Here, we present a multi-resonant nanolaminate SERS substrate designed to achieve RI-insensitive and robust signal enhancement. The vertically stacked metal–insulator–metal architecture provides broadband spectral overlap with both excitation and Raman scattering under dielectric loading, maintaining consistent enhancement across varying RI conditions. We demonstrate label-free bilirubin detection with a highly linear response over 10^−9^ to 10^−4^ M, achieving an R^2^ value of 0.99. Compared with previously reported bilirubin SERS substrates relying mainly on single-resonant plasmonic enhancement, this RI-insensitive design offers improved quantitative reliability under dielectric environmental changes. These results highlight the importance of RI-insensitive SERS design for reliable quantification and provide a general strategy for robust SERS-based biosensing.

## 1. Introduction

Bilirubin is a key clinical biomarker of jaundice and an indicator of hepatobiliary dysfunction and hemolytic disorders [1,2,3,4,5,6]. It is produced during heme catabolism following the breakdown of hemoglobin in red blood cells. In blood, bilirubin is mainly present as poorly water-soluble unconjugated bilirubin, transported predominantly via high-affinity binding to serum albumin [7,8,9,10,11,12]. The albumin-bound form is delivered to the liver, where UGT1A1 catalyzes glucuronidation to generate water-soluble conjugated bilirubin for biliary excretion. Although most unconjugated bilirubin is albumin-bound, a small fraction remains unbound, i.e., free bilirubin. This free fraction is clinically important as it represents a bioavailable species capable of tissue distribution and is a key risk-linked biomarker for bilirubin neurotoxicity and kernicterus, particularly in neonates [10,13,14]. In non-jaundiced children and adults, total bilirubin commonly lies in the sub-mg/dL range, corresponding roughly to single-digit to tens of μM [1,3,15]. In neonates, total bilirubin can increase substantially during physiological jaundice and needs to be closely monitored because elevated bilirubin burden increases the risk of neurotoxicity, motivating rapid and reliable quantification within clinically relevant ranges.

Importantly, free bilirubin exists at far lower concentrations than total bilirubin but is more directly linked to toxicity. Neurophysiological abnormalities have been reported at approximately the 10 nM scale, with cellular toxicity becoming pronounced at higher tens of nM [16,17,18,19,20]. For example, prolonged exposure to 80–90 nM free bilirubin has been associated with substantial cytotoxicity in neural cell models, and the clinical literature reports auditory and neurological abnormalities at 11–17 nM in neonatal settings [19,21,22,23]. These considerations motivate analytical methods capable of sensitive, reproducible detection of bilirubin, especially the low-level free fraction, to enable early intervention.

Numerous approaches have been reported for bilirubin measurement, including diazo-based colorimetry, enzymatic assays, spectrophotometry, chromatography, electrochemical sensing, fluorescence methods, and noninvasive transcutaneous bilirubinometry [2,4,24,25,26]. Routine clinical testing largely relies on diazo and enzymatic assays, which are scalable and automation-compatible but can suffer from matrix-dependent interferences and limited fraction specificity. High-performance liquid chromatography (HPLC) provides fraction-resolved quantification but is typically impractical for rapid and high-throughput workflows. Fluorescence assays enable calibration-based quantification via interactions with a bilirubin probe. Yet, performance can be sensitive to pH and temperature due to solubility and bilirubin–albumin binding equilibria, and it can be degraded by serum autofluorescence. Collectively, these limitations sustain demand for label-free, highly sensitive bilirubin sensing at clinically relevant low concentrations, particularly for free bilirubin.

Surface-enhanced Raman spectroscopy (SERS) amplifies the Raman scattering of molecules located near plasmonic nanostructures via strongly enhanced local electromagnetic fields, called hotspots, produced by surface plasmon resonances [24,27,28,29,30,31]. SERS has been widely applied to chemical and biomedical analysis, as it can deliver label-free molecular fingerprint spectra, enabling identification and quantification without fluorophores or enzymatic amplification, and is compatible with small sample volumes and minimal sample preparation in principle [32,33,34,35,36,37].

For bilirubin detection, SERS offers several advantages over standard readouts, including molecular specificity from vibrational fingerprints that can mitigate spectral cross-talk, label-free operation, potential for multiplexing in complex mixtures, and the prospect of miniaturized, rapid testing platforms. These strengths have motivated multiple bilirubin-related SERS studies, including label-free bilirubin detection in blood- and serum-relevant contexts and the development of engineered substrates with low detection limits. Recent reports include paper-based and hybrid nanostructure substrates for label-free detection of serum bilirubin and for jaundice-related diagnostics, as well as recyclable or defect-engineered substrates that report detection limits down to the 10^−8^ M scale [38,39,40]. These studies collectively support the feasibility of bilirubin SERS and the strong demand for sensitive, label-free approaches. A concise comparison of these representative label-free SERS-based bilirubin sensing platforms is provided in Table S1.

Despite advances in SERS substrate sensitivity and uniformity, quantitative robustness remains challenging since SERS enhancement stems from plasmonic resonance conditions. In general, maximal electromagnetic enhancement is achieved when the plasmonic resonance is well-aligned with the excitation wavelength, meaning that resonance detuning can significantly reduce signal intensity [41,42]. A critical, often under-emphasized factor in detuning is the background refractive index (RI) surrounding the plasmonic nanostructure. Surface plasmon resonances are intrinsically sensitive to the dielectric environment, and increasing the surrounding RI typically redshifts the plasmon resonance. This principle is exploited in localized surface plasmon resonance (LSPR) refractometric sensing [43,44,45,46].

Consequently, if a SERS substrate is designed with a single dominant resonance matched to a specific laser wavelength under one background RI, typically air (*n* = 1), even moderate RI changes at hotspots can shift the resonance away from the excitation wavelength, inducing sample-dependent, off-resonant SERS responses and degraded sensitivity [41,47]. This issue is especially relevant for biological and clinical measurements because the background RI values of most biological samples are not constant; for example, protein- and cell-rich biofluids and whole-blood RI values have been reported across a broad range, reflecting physiological variability [43,48]. In addition, analytes and surrounding biomolecules can perturb the local dielectric environment at hotspots. Bilirubin itself is hydrophobic and has been reported to have a comparatively high RI range of 1.6 to 1.72 [49], suggesting that concentrated bilirubin or bilirubin-rich local regions may further modulate the effective local RI near plasmonic hotspots. Therefore, achieving RI-insensitive, i.e., consistent, SERS becomes important not only across different sample matrices but also across concentration-dependent local environments near hotspots.

In this work, we demonstrate a multi-resonant nanolaminate SERS substrate that delivers RI-insensitive, quantitatively robust enhancement by achieving broadband plasmonic resonance across both the laser excitation and the Stokes-shifted Raman scattering windows under dielectric loading. The nanolaminate architecture, based on vertically stacked metal–insulator–metal (MIM) nanostructures, provides multiple resonances that mitigate resonance detuning when analyte accumulation increases the effective local RI at hotspots. Using simulations and measurements, we verify that this design improves optical tolerance to background RI variations while providing strong enhancement and excellent spatial uniformity, and we further demonstrate label-free quantitative bilirubin detection with a highly linear response over a broad concentration range. More broadly, this multi-resonant strategy offers a general route to improving quantitative SERS in realistic environments where local dielectric properties vary across matrices and with analyte loading. Future integration with standardized sampling and compact readout platforms could facilitate translation toward reliable, point-of-care SERS assays for bilirubin and other clinically relevant biomarkers.

## 2. Materials and Methods

SERS substrate fabrication: A nanowell-patterned polydimethylsiloxane (PDMS) composite stamp (period: 400 nm, diameter: 100 nm, height: 150 nm) was fabricated from a nanopillar-patterned silicon master via soft lithography. Using this stamp, nanopillar arrays of a UV-curable polyurethane (PU) were formed on a flexible polyester (PET) film, followed by UV curing and subsequent thermal curing. Alternating Au and silicon dioxide multilayers were then deposited by electron-beam evaporation, comprising four Au layers (30 nm each) and three silicon dioxide layers (6, 8, and 12 nm from bottom to top). To expose the nanogap-based plasmonic hotspots, the silicon dioxide layers were partially etched using buffered oxide etchant (10:1).

Finite-difference time-domain (FDTD) simulation: Optical simulations were carried out using the FDTD solution in Ansys Lumerical (version 2020 R2.1, Vancouver, BC, Canada). A mesh size of 2 nm was applied in all directions. The optical constants of gold were taken from Johnson and Christy. The Bloch boundary condition was applied in the x- and y-directions with a periodicity of 400 nm, and the perfectly matched layer boundary condition was applied in the z-direction. The refractive indices of silicon dioxide and PU were set to 1.5 and 1.56, respectively.

SERS enhancement factor (EF) calculation: The EF was calculated using the equation EF = (*I_SERS_*/*I_Raman_*) × (*N_Raman_*/*N_SERS_*), where *I_SERS_* and *I_Raman_* represent the measured SERS and neat Raman intensities, respectively, and *N_SERS_* and *N_Raman_* denote the number of molecules contributing to each signal. *N_SERS_* was estimated as *N_SERS_* = *SA* × *ρ_SERS_*, where SA is the illuminated surface area on the SERS substrate and *ρ_SERS_* is the molecular packing density of benzenethiol (6.8 × 10^14^ molecules/cm^2^). *N_Raman_* was determined using *N_Raman_* = *A* × *d_eff_* × *ρ_Raman_*, where A is the laser-illuminated area, *d_eff_* is the effective focal depth, and *ρ_Raman_* is the molecular density of neat benzenethiol (5.9 × 10^21^ molecules/cm^3^). The *d_eff_* value was experimentally measured using a bare silicon wafer by adjusting the objective focus in incremental steps.

Sample preparation: Bilirubin powder was first dissolved in dimethyl sulfoxide to prepare a stock solution, which was then serially diluted with deionized water to obtain bilirubin solutions ranging from 10^−4^ to 10^−9^ M. Considering the light sensitivity of bilirubin, the bilirubin powder and prepared solutions were protected from direct light before use. For SERS measurements, 0.2 μL of each bilirubin solution was drop-cast onto the nanolaminate SERS substrate and allowed to dry under ambient conditions prior to spectral acquisition. After drying, Raman spectra were acquired from the dried residue region of each droplet. The same droplet volume, drop-casting procedure, drying condition, and Raman acquisition condition were applied to all bilirubin concentrations.

SERS measurement: We used a confocal Raman microscope (Thermo Fisher Scientific, Waltham, MA, USA) under 785 nm laser excitation in a backscattering geometry, as schematically illustrated in Figure S2. A 50× objective lens (NA = 0.75) was used with a laser power of 1 mW and an integration time of 1 s. The collected Raman signals were spectrally dispersed and detected using a CCD detector. The system was calibrated using the silicon peak.

## 3. Results

**Figure 1:**
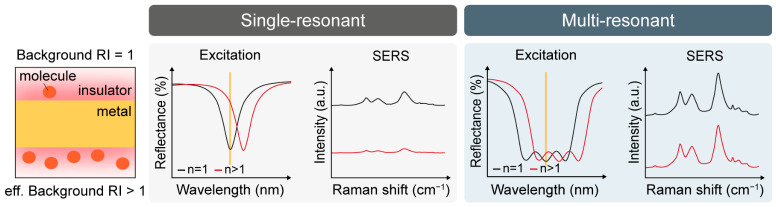
Schematic illustration comparing the refractive index (RI)-sensitive single-resonant mode and the RI-insensitive multi-resonant mode under excitation and varying background RI.

Figure 1 schematically illustrates how the background RI governs resonance matching and, consequently, the reliability of SERS measurements. In single-resonant substrates (left), the nanostructure is typically designed so that its plasmonic resonance overlaps the excitation wavelength under a nominal environment (*n* = 1), maximizing near-field intensity at hotspots. During measurement, however, analyte molecules can occupy the local hotspot region, increasing the local dielectric loading and thus the effective RI (*n* > 1). As plasmonic resonances are highly sensitive to the surrounding dielectric constant, this increase in RI induces a redshift of the resonance. The excitation line then becomes off-resonant, leading to a substantial reduction in local field enhancement and Raman amplification.

As a result, the measured SERS intensity can decrease even when more molecules are present at the same hotspot, leading to a distorted concentration–signal relationship and reduced quantitative reliability. To mitigate this intrinsic RI sensitivity, we employ a multi-resonant SERS strategy (right) that supports multiple resonances distributed around both the excitation wavelength and the Stokes-shifted Raman scattering range. When dielectric loading increases the effective RI and redshifts the plasmon resonance, these multiple resonances shift together but maintain spectral overlap with the fixed excitation, as the enhancement is not confined to a single narrow resonance. In other words, the probability that the excitation remains close to an enhancement maximum is much higher. Hence, the near-field intensity and thus the SERS signal remain comparatively stable under local RI perturbations. Consequently, the multi-resonant design delivers RI-insensitive, consistent SERS performance, enabling more robust quantification in environments with local RI variations.

**Figure 2:**
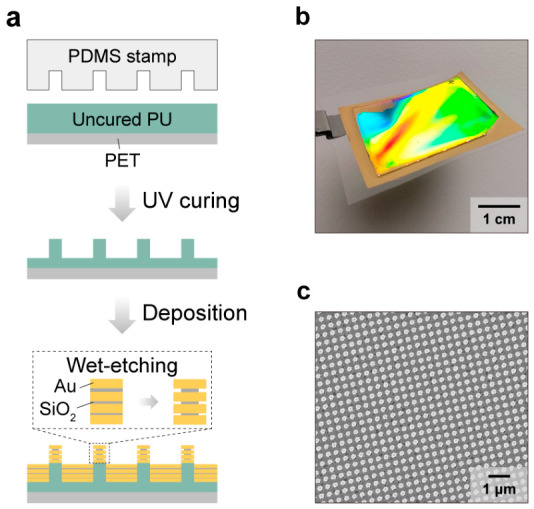
SERS substrate with a nanolaminate structure: (**a**) Schematic illustration of the nanolaminate fabrication process. (**b**) Photography and (**c**) top-view SEM images of the fabricated nanolaminate SERS substrate.

Figure 2a shows the fabrication steps for the multi-resonant nanolaminate SERS substrates. Briefly, a nanohole-patterned polydimethylsiloxane (PDMS) stamp was prepared by soft lithography based on a nanopillar-patterned silicon wafer. Polyurethane (PU) was molded onto a polyester (PET) film via nanoimprint lithography, yielding periodic nanopillar arrays. Then, alternating layers of gold and silicon dioxide were deposited without rotation, and wet etching was performed to partially open nanogaps in the MIM structures [50].

Figure 2b shows an optical image of the substrate. A clear diffraction pattern confirms the periodic nanostructures over a large area. Figure 2c shows a representative scanning electron microscope (SEM) image and further confirms the periodic nanostructures with a 400 nm periodicity. Unlike conventional single-resonant SERS substrates, the nanolaminate structure provides multiple plasmon resonances, and their resonance wavelengths can be readily tuned by adjusting the thickness of the insulating layers [41]. Increasing the insulator thickness weakens plasmonic hybridization between the coupled electric dipoles of each nanodisk, lowering the mode energy and thereby redshifting the resonance wavelength. Moreover, the presence of multiple MIM structures increases the hotspot density per unit nanostructure, enhancing overall sensitivity. Collectively, these features can enable a more consistent SERS response under varying background RI, improving measurement robustness and quantitative reliability.

**Figure 3:**
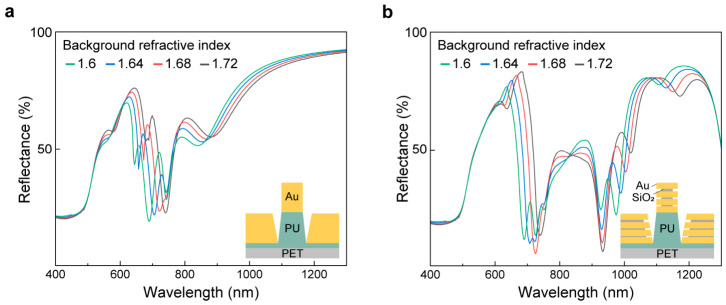
Optical properties of SERS substrates under different background RIs. FDTD-calculated reflectance spectra of SERS substrates (**a**) without nanolaminate and (**b**) with nanolaminate structure, with the background RI varying from 1.60 to 1.72, corresponding to the effective RI of bilirubin.

To assess the background RI sensitivity of nanolaminate SERS substrates, we performed finite-difference time-domain (FDTD) simulations and calculated reflectance spectra for the non-nanolaminate control and the nanolaminate substrate while varying the background RI from 1.60 to 1.72 (Figure 3). The RI range was selected to approximately represent the effective dielectric environments that may develop at plasmonic hotspots during bilirubin enrichment with different local packing densities, thereby providing a realistic perturbation window for evaluating resonance detuning.

As shown in Figure 3a, the non-nanolaminate structure exhibits only a few resonance features across the visible-to-near-infrared window, with two prominent dips. Increasing the background RI causes a clear redshift of these resonances, consistent with dielectric loading, where an increased surrounding permittivity increases the effective optical path and shifts the resonance condition to longer wavelengths. Because the non-nanolaminate substrate is designed to place its primary resonance near the 785 nm excitation under an air background (Figure S1), even moderate RI increases shift the resonance away from the fixed laser line, increasing the probability of off-resonant excitation and reducing electromagnetic enhancement. This result implies that the SERS performance of a single-resonant substrate can be significantly modulated by RI fluctuations that accompany hotspot occupation.

In contrast, the nanolaminate structure (Figure 3b) reveals a substantially richer, broader resonant response, with multiple resonances and up to six dips distributed from the visible to the near-infrared. Although these modes also redshift with increasing RI, their dense spectral distribution provides broadband resonance coverage, which prevents resonance detuning. Notably, the broad plasmonic response of the nanolaminate substrate can maintain spectral overlap not only with the excitation wavelength (785 nm) but also with the Stokes-shifted Raman scattering region, which is critical for maximizing the overall SERS enhancement through effective coupling at both the pump and emission wavelengths. As the background RI increases, the collective presence of multiple modes increases the likelihood that at least one resonance remains near the excitation wavelength while simultaneously providing enhanced near-field support across the Raman-shifted wavelengths, thereby stabilizing the effective SERS performance under dielectric perturbations. Collectively, the reflectance comparison highlights a key advantage of the nanolaminate architecture. Changes in the local dielectric environment can readily detune the single-resonant design, whereas the nanolaminate design offers broadband resonance coverage that is inherently more tolerant to background RI variations. This broadband resonance coverage helps maintain spectral overlap with the excitation and Raman scattering windows under RI variation, thereby reducing resonance-detuning-induced variation in SERS enhancement. This optical robustness is expected to translate into more stable and consistent SERS enhancement and improved quantitative reliability, particularly in sensing scenarios where the effective RI evolves during analyte adsorption and concentration-dependent hotspot loading.

**Figure 4:**
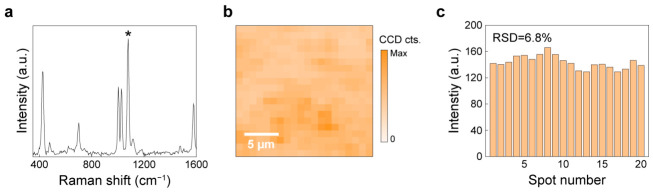
Performance of nanolaminate SERS substrate: (**a**) The average SERS spectra of BZT. The asterisk (*) indicates the 1078 cm^−1^ peak. (**b**) 2D Raman image of 20 μm × 20 μm for the BZT peak at 1078 cm^−1^. (**c**) Uniformity assessment of nanolaminate structure based on 20 measurement spots.

To evaluate the intrinsic SERS performance of the nanolaminate substrates, we first employed a benzenethiol (BZT) self-assembled monolayer as a standard non-resonant Raman probe, widely used to evaluate SERS sensitivity. Figure 4a shows the averaged SERS spectrum of BZT, where the characteristic vibrational signatures are clearly resolved, including prominent bands near 1000 cm^−1^, 1078 cm^−1^, and 1575 cm^−1^, corresponding to carbon–carbon–carbon (C–C–C) ring in-plane deformation vibration, the C–C–C ring in-plane breathing mode coupled with the carbon–sulfur (C–S) stretching mode, and the C–S stretching mode, respectively [51].

Using the 1078 cm^−1^ peak, we calculated the SERS enhancement factor (EF) to be on the order of 10^7^ using the widely adopted formula [50,52]. As shown in Figure 4b, we further assessed the spatial uniformity over a 20 μm × 20 μm region, demonstrating a largely homogeneous signal distribution without isolated, extreme hotspots. To quantify spot-to-spot variation, we extracted the peak intensity at 1078 cm^−1^ from 20 representative spots within the mapped region. Figure 4c shows that the intensity variations yield a low relative standard deviation (RSD) of 6.8%, confirming excellent hotspot uniformity across the substrate. These results establish that the nanolaminate SERS substrate can provide high SERS performance, combining strong enhancement with great uniformity, a prerequisite for reliable quantitative measurements in subsequent bilirubin experiments.

**Figure 5:**
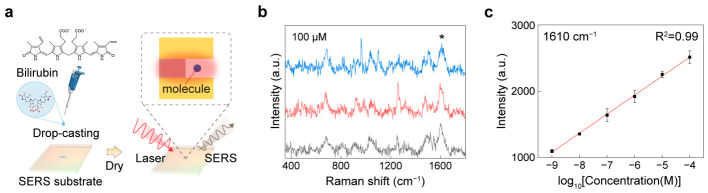
SERS detection of bilirubin: (**a**) Schematic illustration of the label-free SERS measurement configuration for bilirubin detection using a nanolaminate SERS substrate. (**b**) Representative SERS spectra of 100 µM bilirubin measured at three different spots. The asterisk (*) indicates the 1610 cm^−1^ peak. (**c**) Calibration curve showing the linear relationship between the logarithm of bilirubin concentration and the SERS intensity at 1610 cm^−1^. Error bars represent the standard deviation from three measurements acquired at different spots.

To demonstrate label-free quantitative detection of bilirubin using the nanolaminate SERS substrate, we adopted a simple drop-casting workflow that is commonly used for benchmarking small-molecule sensing on solid substrates (Figure 5a). Bilirubin solutions were prepared at a series of concentrations and then drop-cast onto the SERS substrate. After solvent evaporation, bilirubin molecules concentrate at and near plasmonic hotspots, minimizing additional chemical labeling or complex pretreatment steps and providing a direct assessment of how reliably the substrate converts analyte loading into a measurable SERS response. Figure 5b shows representative SERS spectra acquired from 100 µM bilirubin samples, illustrating that bilirubin produces reproducible vibrational signatures on the nanolaminate substrates. More specifically, we can observe bilirubin characteristic peaks of 687, 1267, and 1610 cm^−1^, corresponding to the ring deformation in the pyrrole ring, lactam ring C–C stretching with N–H bending, and the C=C stretching in the five-membered ring, respectively [53,54]. To further support the low-concentration detection result, the SERS spectra of 10^−9^ M bilirubin measured from three different spots, together with the corresponding averaged spectrum, are provided in Figure S3.

For quantitative analysis, we extracted the peak intensity at 1610 cm^−1^ from each spectrum and plotted it as a function of concentration (Figure 5c). The resulting calibration curve exhibits excellent linearity over a broad dynamic range spanning 10^−9^ to 10^−4^ M, with a strong coefficient of determination (R^2^ = 0.99). The error bars indicate the standard deviation across measurements, demonstrating good reproducibility of the intensity readout at a fixed Raman marker band. This wide-range linear response is advantageous for practical sensing because bilirubin levels of interest can vary substantially depending on physiological state and sample conditions, and robust quantification requires both sensitivity at low concentrations and stability at higher loading.

Collectively, these results demonstrate that the nanolaminate SERS substrate enables direct, label-free, concentration-dependent bilirubin detection with clear spectral fingerprints and highly linear quantitative performance over five orders of magnitude in concentration. Furthermore, these results indicate that the nanolaminate structure can provide RI-insensitive SERS, a key requirement for translating SERS to realistic sensing scenarios where analytes and local environments exhibit variable background RI values. In this study, bilirubin measurements in a simple aqueous solution were used to demonstrate the RI-insensitive SERS enhancement strategy of the nanolaminate substrate. For improved clinical relevance, further validation in albumin-containing buffers, serum-like media, or spiked biological samples can be used, and the matrix effect should be considered.

## 4. Conclusions

This work demonstrates that background RI variations in the hotspot environment can be a major source of instability in quantitative SERS, as they shift plasmonic resonances away from a fixed excitation wavelength and reduce electromagnetic enhancement. We addressed this issue by developing a multi-resonant nanolaminate SERS substrate that provides broadband resonance coverage near the 785 nm excitation wavelength and across the Stokes-shifted Raman scattering region. This broadband response enables the substrate to preserve SERS performance even when analyte accumulation increases the effective local RI at hotspots. Optical simulations over a bilirubin-relevant RI range showed that the nanolaminate architecture supports a much richer resonance distribution than the non-nanolaminate control, thereby improving tolerance to dielectric loading and reducing resonance detuning. SERS measurements using a BZT self-assembled monolayer confirmed that the substrate provides strong enhancement and excellent spatial uniformity.

Based on this high SERS performance, we achieved label-free quantitative bilirubin detection and obtained a highly linear calibration over 10^−9^ to 10^−4^ M. Beyond bilirubin, the central implication is that multi-resonant plasmonic architectures can enable quantitative SERS performance despite unavoidable dielectric variability in realistic samples. This is particularly relevant for biofluids and tissue-derived specimens in which RI varies with protein content, cellular components, viscosity, and drying or concentration effects, and for hydrophobic or high RI analytes that locally perturb the dielectric environment at the interface.

The nanolaminate design principle provides an approach to improve measurement robustness for SERS assays targeting diverse small molecules, metabolites, and biomarkers that are otherwise challenging to quantify reliably on single-resonant substrates. Future work can translate this platform into clinically actionable formats by moving from dried drop-casting to controlled sampling workflows, including microfluidic delivery and standardization of incubation and washing steps. The high uniformity of the substrate also supports area-averaged readout and automated mapping, which can reduce operator dependence and improve inter-sample variation. Collectively, these directions position multi-resonant SERS substrates as a practical foundation for robust, quantitative SERS-based diagnostics in complex, variable biological environments.

## Data Availability

The data presented in this study are available upon reasonable request from the corresponding author.

## Supplementary Materials

The following supporting information can be downloaded at: https://www.mdpi.com/article/10.3390/bios16050282/s1, Table S1: Representative label-free SERS-based bilirubin sensing platforms and their key sensing strategies; Figure S1: FDTD-calculated reflectance spectra of SERS substrates (a) without nanolaminate and (b) with nanolaminate structure at a background RI of 1.00 (air) under 785 nm excitation; Figure S2: Schematic illustration of the confocal Raman microscope setup used for SERS measurements in a backscattering geometry; Figure S3: SERS spectra of 10^−9^ M bilirubin measured from three different spots and the corresponding averaged spectrum.

## Abbreviations

The following abbreviations are used in this manuscript:

SERS	Surface-enhanced Raman spectroscopy
RI	Refractive index
HPLC	High-performance liquid chromatography
LSPR	Localized surface plasmon resonance
MIM	Metal–insulator–metal
PDMS	Polydimethylsiloxane
PU	Polyurethane
PET	Polyester
FDTD	Finite-difference time domain
EF	Enhancement factor
SEM	Scanning electron microscope
BZT	Benzenethiol
C–C–C	Carbon–carbon–carbon
C–S	Carbon–sulfur
RSD	Relative standard deviation
