# Supplementary material for: Label-Free Quantification of Bilirubin Using a Refractive Index-Insensitive Nanolaminate SERS Substrate
Source: Biosensors (Basel). 2026 May 14;16(5):282. doi: 10.3390/bios16050282 (PMC13204365; doi:10.3390/bios16050282)
Supplement: Supplementary file 1 [file biosensors-16-00282-s001.zip › biosensors-4270659-supplementary.pdf]

biosensors

MDPI

Supporting Information

Label-Free Quantification of Bilirubin Using a Refractive Index-Insensitive Nanolaminate SERS Substrate

Jiwon Yun [1], Inyoung Kim [2] and Wonil Nam [1,3,*]

1 Department of Intelligent Robotics Engineering, Pukyong National University, Busan 48513, Republic of Korea; 202112150@pukyong.ac.kr
2 Department of Statistics, Virginia Polytechnic Institute and State University, Blacksburg, VA 24061, USA; inyoungk@vt.edu
3 Department of Electronic Engineering, Pukyong National University, Busan 48513, Republic of Korea; nam@pknu.ac.kr
* Correspondence: nam@pknu.ac.kr; Tel.: +82-051-629-6217

Table S1. Representative label-free SERS-based bilirubin sensing platforms and their key sensing strategies.

SERS platform	Sample matrix	Detection performance	Key strategy	Reference
Graphene oxide–Au nanostar hybrid paper-based SERS biosensor	Free bilirubin in blood serum	Linear ranges: 5.0–150 μM and 150–500 μM; LOD: 0.436 μM	Enrichment-assisted serum bilirubin detection	[Ref. 38] Biosensors and Bioelectronics, 2019, 145, 111713
Boron nitride-modified nanorod array SERS substrate	Bilirubin in blood / spiked blood samples	LOD: 1.4 × 10^{-8} M; RSD: <15% R^2=0.97	Improved adsorption, stability, and reusability	[Ref. 39] Sensors and Actuators B: Chemical, 2021, 334, 129634
Functionalized MoS_2 SERS substrate	Bilirubin solution with dextrose and phosphate interferents	Linear range: 10^{-3}–10^{-9} M; LOD: 10^{-8} M	Selective detection against interferents	[Ref. 40] Microchimica Acta, 2023, 190(3), 83

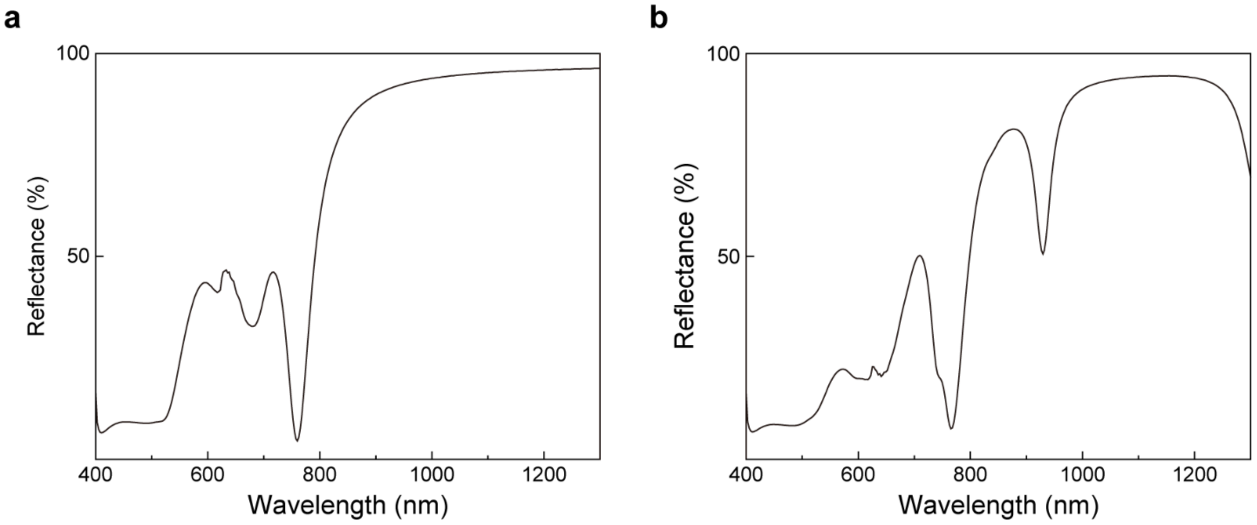

Figure S1. FDTD-calculated reflectance spectra of SERS substrates (a) without nanolaminate and (b) with nanolaminate at a background RI of 1.00 (air) under 785 nm excitation.

 https://doi.org/10.3390/bios16050282

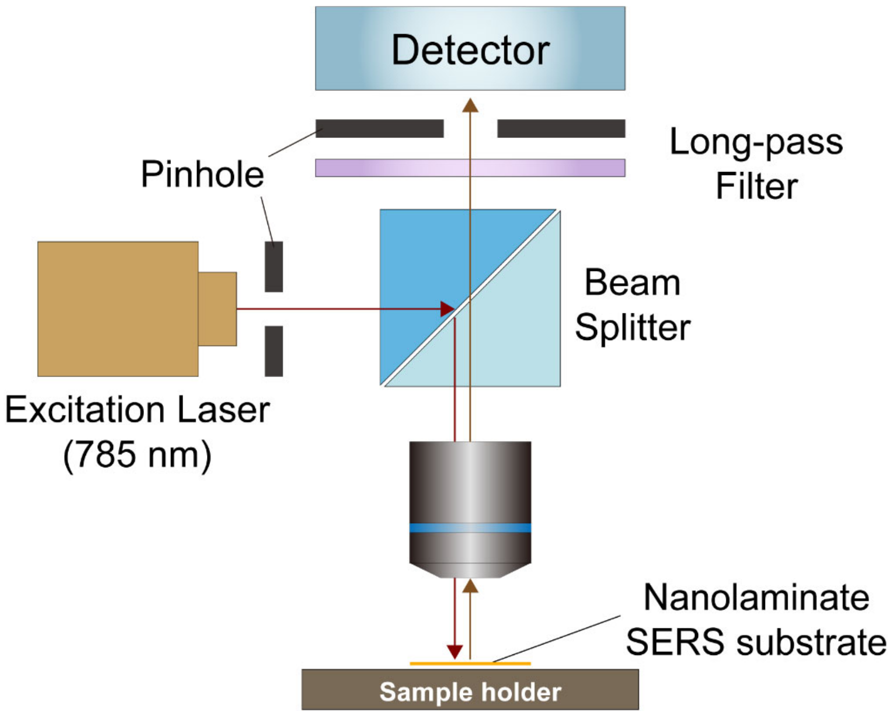

Figure S2. Schematic illustration of the confocal Raman microscope setup used for SERS measurements in a backscattering geometry.

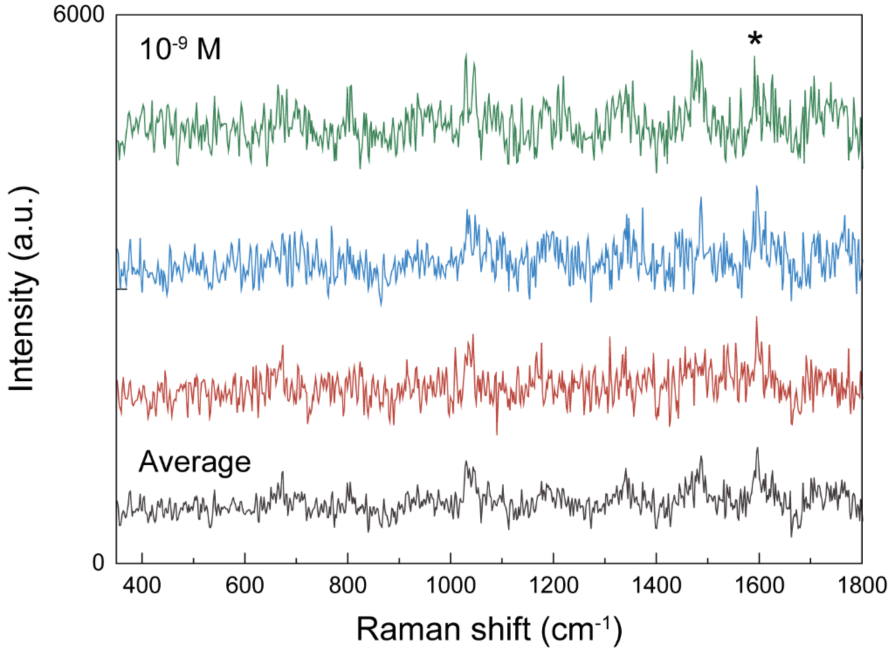

Figure S3. SERS spectra of 10^{-9} M bilirubin measured from three different spots and the corresponding averaged spectrum.

https://doi.org/10.3390/bios16050282
